# Detection and comparison of tumor cell-associated microbiota from different compartments of colorectal cancer

**DOI:** 10.3389/fonc.2024.1374769

**Published:** 2024-05-21

**Authors:** Yanzhen Zuo, Yanjie Lu, Jiayu Pang, Shunkang Jin, Xinyu Zhang, Enhong Zhao, Yuhong Li

**Affiliations:** ^1^ Cancer Research Laboratory, Chengde Medical College, Chengde, Hebei, China; ^2^ Department of Gastrointestinal Surgery, Affiliated Hospital of Chengde Medical College, Chengde, Hebei, China

**Keywords:** colorectal cancer, tumor cell-associated microbiota, microbial populations, contamination, enzymatic digestion

## Abstract

**Introduction:**

Intratumoral microbes play an important role in the development of colorectal cancer (CRC). However, studying intratumoral microbes in CRC faces technical challenges, as tumor microbe communities are often contaminated by fecal microbes due to the structure of the gut folds and villi. The present study aimed to develop a new method for isolating tumor cell-associated microbiota and comparing microbial populations from different compartments.

**Materials and methods:**

The distribution of intestinal bacteria was detected using immunohistochemistry combined with 5R-16s rRNA gene sequencing to explore the effects of the sampling site and number of washes on the detection of microbiota. The 5R-16s rRNA gene sequencing was performed using 44 samples from 11 patients with CRC, including CRC tumor tissues (TT), normal tissues adjacent to CRC (NT), tumor cells (TC), and normal cells (NC). TC and NC were obtained from the TT and NT using an enzymatic digestion method. The microbiota and their potential functions in the four groups were analyzed and compared to determine the differential microbiota related to CRC.

**Results:**

Bacteria were mainly distributed in the feces covering intestinal tissues and in the epithelial cells and macrophages within the tissues. Different sampling sites and number of washes led to detection of different microbiota distributions. Although the cleaning method could be controlled, sampling sites varied and led to different microbiota distributions. The phyla of Firmicutes and Bacteroidetes were highly abundant in the conventionally used tissue samples, whereas Proteobacteria was the most abundant phyla in the cell samples isolated with the new method (i.e., after cell enzymatic hydrolysis). Detection of CRC cell-associated microbiota using a cell enzymatic digestion method showed that some bacteria, such as *Fusobacterium, Eikenella, Shewanella*, and *Listeria*, were more abundant in TT than NT, whereas the abundance of *Akkermansia* was lower in TT than NT. The tumor/normal ratios of some bacteria, such as *Gemella, Escherichia, Shigella*, and *Blautia*, were different between the cell and tissue samples.

**Conclusion:**

The cell enzymatic digestion method reduced fecal bacterial contamination, enabling low biomass intratumoral microbiota to be detected and allowing prediction of bacterial distributions.

## 1 Introduction

Colorectal cancer (CRC) is one of the most common malignancies of the digestive tract. According to the 2020 Global Cancer Statistics report, CRC has the third highest incidence and the second highest mortality in the world (1). The largest number of microbes in the human body exist in the gastrointestinal tract, which comprise a complex ecosystem known as the gut microbiome. With the development of new sequencing technology and bioinformatics, it is possible to further study the composition, metabolism, and metagenome of intestinal microecology (2). While many studies have revealed a strong relationship between gut microbiota and CRC, most have focused on fecal microbiota and their metabolites (3). Due to the complexity of fecal microbiota and the multi-factor regulation of their interactions with the intestinal mucosal epithelium, as well as the fact that most of the bacteria are “passing bacteria” rather than “colonizing bacteria,” exploring the direct evidence and biological behavior of bacterial enrichment and their invasion of tumor tissues and cells has become a new research direction in this field. In recent years, some studies have focused on tumor-associated microbiota. Tumor-associated microbiota are intrinsic components of the tumor microenvironment in human cancer types (4–6). Intratumoral microbiota are integral tumor components that play critical roles in shaping the tumor microenvironment (7, 8). Intratumoral bacteria are mostly intracellular and are present in both cancer and immune cells; each tumor type has a distinct microbiome composition (5). The functions of intratumoral bacteria are associated with the tumor type/subtype and their responses to immunotherapy (5). Cancer cells infected with bacteria invade the surrounding environment as single cells and recruit myeloid cells to the bacterial regions. Intratumoral microbiota are mainly distributed and enriched in microniches, influencing immune and epithelial cell functions that promote cancer progression (4). Tumor-associated bacteria play a crucial and direct role in inducing tumorigenesis, changing cell metabolism, promoting tumor metastasis, and preventing immune surveillance (9). Investigating tumor-associated microbiota may aid the discovery of novel treatment options for patients with cancer.

**Figure 1:**
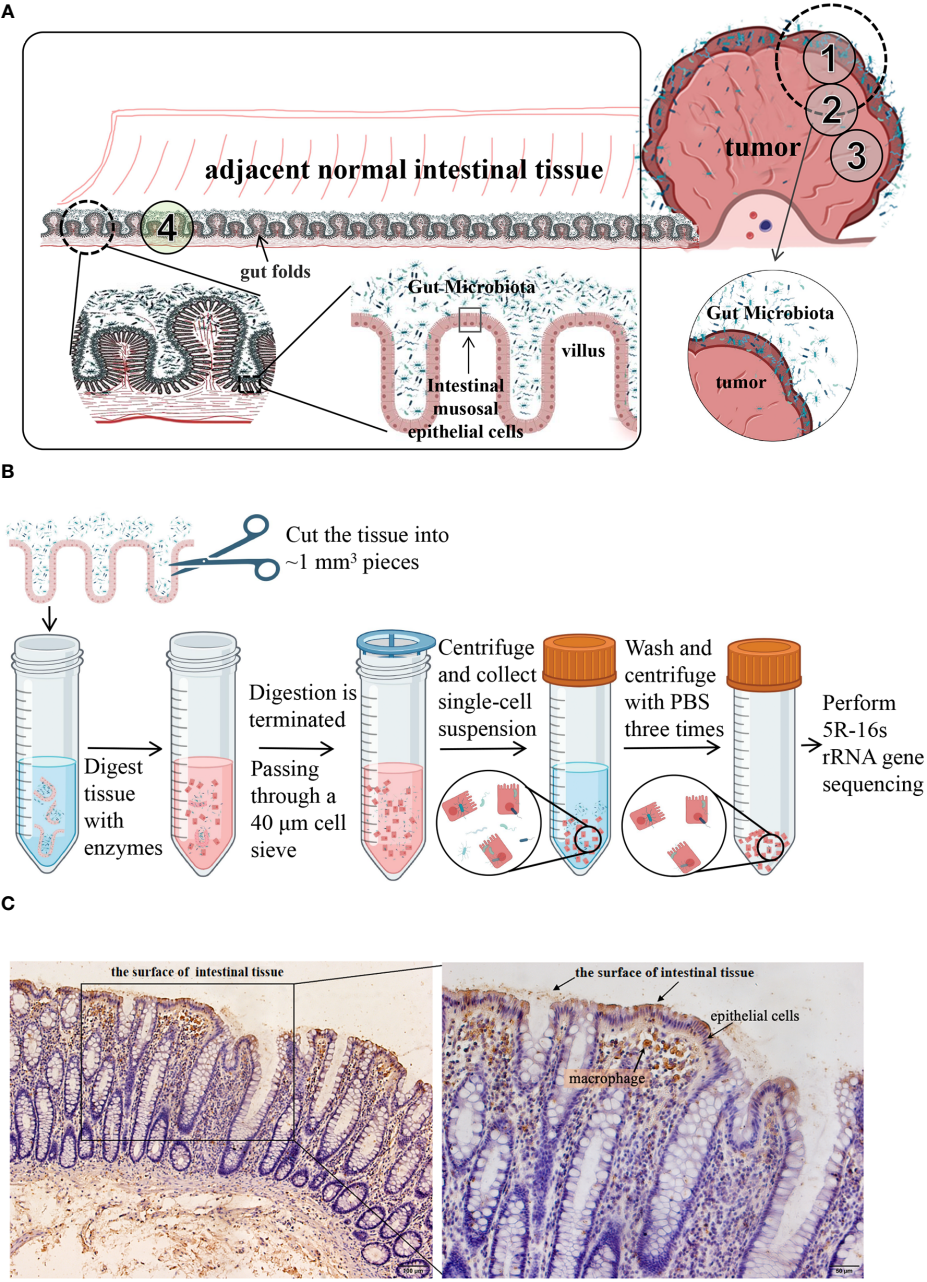
**(A)** Schematic diagram of the distribution of colorectal and intestinal microbes. CRC tumors are inherently contaminated by fecal microbes owing to the structure of the gut folds and villi. ①, ②, ③, and ④ are schematic diagrams of the sampling sites. CRC tumor tissues and the adjacent normal tissues from 11 patients were taken as shown in ① through ④. **(B)** a schematic diagram of isolation process of cell-associated microbiota. **(C)** Immunohistochemical analysis of LPS. LPS staining was mainly observed in the feces from the surface of intestinal tissues and in epithelial cells and macrophages within the tissues. LPS, lipopolysaccharide.

However, unlike other solid tumors, such as breast cancer, which have no direct connection with the external environment, CRC tumors are inherently contaminated by fecal microbes owing to the structure of the gut folds and villi (**Figure 1A**). Preventing contamination with and detection of tumor-associated microbiota are challenging due to technical difficulties. In the present study, we explored different methods for detecting the distribution of gut microbes and established a new method for isolating the tumor cell-associated microbiota of CRC. The microbiota and their potential functions were analyzed and compared using different methods, and the tumor cell-associated microbiota of CRC was obtained.

## 2 Materials and methods

This study was performed in accordance with the guidelines of the 1975 Declaration of Helsinki, revised in 1993. All participants provided informed consent and the study was approved by the Medical Ethics Committee of Chengde Medical University (No. 202215).

### 2.1 Patients

CRC tumor samples were obtained from patients who underwent surgery at the Affiliated Hospital of Chengde Medical College, Chengde City, Hebei Province, China, between September and December of 2022. The inclusion criteria were: (1) patients who had been diagnosed with CRC; (2) no previous history of colorectal surgery; (3) no use of antibiotics or corticosteroids or probiotics one month prior to the surgery; (4) no familial adenomatous polyposis or hereditary nonpolyposis CRC; no inflammatory bowel disease, metabolic diseases (such as diabetes, obesity, hyperlipidemia), infectious diseases, severe liver disease, kidney diseases, or immunodeficiency; (5) no special dietary habits; and (6) patients who volunteered to participate in the study. The exclusion criteria were as follows: (1) pregnancy or lactation, (2) previous cancer diagnosis, (3) patients who had received neoadjuvant chemoradiotherapy, and (4) unwillingness to participate in the study. Based on the aforementioned inclusion and exclusion criteria, 12 patients with CRC were included in this study.

### 2.2 Colorectal cancer and control samples

Samples were collected from the 12 patients through surgery. Following radical resection of CRC, the tumor tissue (TT) and adjacent normal intestinal tissue (NT, more than 5 cm distant from the cancer) were separately cut with a sterile knife. The samples were placed in sterile tubes, immediately transported to the laboratory in an icebox, and washed three times with sterile saline. A portion of each sample was taken and fixed in 4% paraformaldehyde separately for histological and immunohistochemical (IHC) staining, and the remaining tissue was used in the following steps. TT and NT from 11 patients were taken as shown in **Figure 1A** ① through ④. They all contained areas of contact with feces and duplicate samples were taken, one being frozen in liquid nitrogen for subsequent 5R-16s rRNA gene sequencing and another being used to isolate tumor cells (TC) or normal cells (NC) as described below. The sampling of the twelfth patient was slightly different from the 11 patients mentioned above. As shown in **Figure 1A**, TT from the twelfth patient was sampled from three areas (①, ②, and ③), part ① containing fecal bacteria adhering to the surface of the tumor tissue while part ② and ③ containing no areas in contact with fecal bacteria. During the surgery, a pathologist identified the mucosal part and used a surgical knife to remove it. Part ③ was washed three more times with sterile saline. Three NT samples were taken as shown in part ④, one being frozen in liquid nitrogen for subsequent 5R-16s rRNA gene sequencing, the second being washed three more times, while the third was washed six times with sterile saline. Six samples from the twelfth patient were frozen in liquid nitrogen for subsequent 5R-16s rRNA gene sequencing. All tissues were sampled in a sterile biosafety cabinet. Each sample had a wet weight of at least 200 mg. An empty tube was set up as an environmental control tube in parallel to the experimental tubes during sample collection procedures, with a total of 3 environmental control tubes used.

### 2.3 Histological and IHC staining

The paraffin-embedded tissues were cut into 4-μm thick sections. After deparaffinization and hydration, a representative section of each sample was stained with hematoxylin and eosin to confirm the pathological diagnosis of the tumor and normal tissues. Consecutive tissue sections were subjected to IHC staining. Antigen retrieval was performed by boiling the slides in sodium citrate solution. Then, 3% H_2_O_2_ was used to eliminate the endogenous peroxidase activity. Next, tissue sections were incubated with mouse anti-*E. coli* lipopolysaccharide (LPS) antibodies (1:200 dilution; Abcam, cat# ab35654) overnight at 4℃ followed by incubation with horseradish peroxidase-conjugated anti-mouse secondary antibodies. Color development was performed using diaminobenzidine. The tissue sections were counterstained with hematoxylin, dehydrated, mounted, and observed under a light microscope.

### 2.4 Isolation of tumor cell-associated microbiota of CRC

Tissues from 11 patients (see section 2.2) was enzymatically processed to obtain single cells (TC and NC), respectively. As shown in **Figure 1B**, the tissue was cut into ~1 mm^3^ pieces and digested in a 4 mL solution with 1 mg/mL dispase (CAS: 42613-33-2, D6430, Solarbio) and 1 mg/mL collagenase (CAS: 9001-12-1, C8160, Solarbio) for 30 min at 37°C. After enzymatic digestion, the samples were diluted in Dulbecco’s modified Eagle’s medium (DMEM) containing 20% fetal bovine serum. The cell suspension was filtered through a 40-μm cell strainer (BD 352340) to collect the single-cell suspension. After centrifugation at 300 x g for 5 min, the supernatant was discarded. The cell pellet was washed three times with 5 mL phosphate-buffered saline. Finally, the cells were resuspended in 500 µL serum-free DMEM and frozen in liquid nitrogen for subsequent 5R-16s rRNA gene sequencing.

### 2.5 5R-16s rRNA gene sequencing

5R-16s rRNA gene sequencing was performed as described previously (10) (5). DNA from frozen samples were extracted with the CTAB method using kit DP302-02 (TianGen, Beijing, China) and were quantified using Qubit (Invitrogen, USA). 16S rRNA gene amplification and sequencing were performed by amplifying five (V2, V3, V5, V6, and V8) regions of the 16S rRNA gene in a multiplex (Primer sequences are described in **Supplementary Material S1**). PCR products were purified with AMPure XT beads (Beckman Coulter Genomics, Danvers, MA, USA) and quantified with Qubit (Invitrogen, USA). Purified PCR products were evaluated using an Agilent 2100 Bioanalyzer (Agilent, USA) and Illumina (Kapa Biosciences, Woburn, MA, USA) library quantification kit. The qualified library concentration should be above 2 nM. Sequencing was performed using NovaSeq 6000 sequencer with NovaSeq 6000 SP Reagent Kit (500 cycles). The Short Multiple Regions Framework (11) was used to analyze the sequences of the five amplified regions for bacterial taxonomic identification. We used the Greengenes database for this project (ver. May 2013). To reduce the effect of low-abundance noise on subsequent analyses, the number of sequence reads per sample was normalized to remove samples with less than 1000 total reads (including negative controls) and bacterial data with a relative abundance of less than 10^-4^. A total of 19 empty tubes were set up as negative control tubes during the DNA isolation and sequencing procedures, which were used to exclude contaminated bacteria together with the 3 environmental control tubes. Considering the low load biomass of the samples, bacteria introduced from the sampling environment and sequencing experiments will cause big interference to the microbiota of the samples, thus environmental controls (an empty tube was set up in parallel to the experimental tubes during sample collection procedures) and negative controls (an empty tube set up in parallel to the experimental tubes during DNA isolation and sequencing procedures) were set up. Based on the prevalence of bacteria in the control tubes, the contaminating bacteria at the sampling end and the sequencing end were determined. In this project, more than 50% prevalence was set as the threshold for identification of contaminating bacteria, that is, when ≥ 2 of the 3 environmental control tubes or ≥ 9 of the 19 negative controls showed the species, the species was considered as contaminated bacteria and was excluded from analyses. After removing contaminating bacteria, diversity analysis and differential flora identification within and between groups were performed based on the filtered flora data.

### 2.6 Data analysis

In the alpha diversity analysis, species richness and evenness were measured, as well as sequencing depth. The Chao1 and Observed_species indices reflected the species richness of the samples. Good coverage reflected the flora coverage of the sample. The Simpson and Shannon indices were mainly used to reflect the richness and evenness of species. Beta diversity analysis was performed to analyze species differences between groups. Principal component analysis (PCA), principal coordinate analysis (PCoA), non-metric multidimensional scaling (NMDS), analysis of similarities (ANOSIM) and other methods were utilized to observe differences between samples. The ratios of the abundance of each bacterial species to the abundance of all species at each taxonomic level (relative abundance) were calculated, and stacked bar charts of bacterial species distribution in different samples (or groups) at each taxonomic level were drawn to analyze and compare the changes in bacterial composition between different groups. A Venn diagram was used to analyze the number of common and unique species across multiple groups at each taxonomic level to visually show the similarity and specificity of species composition in different groups. Linear discriminant analysis effect size (LEfSe) (12) was used to analyze differences in the microbiota compositions. In this study, the threshold of the LEfSe analysis was set at a Linear Discriminate Analysis (LDA) value > 3, *p* < 0.05. The results are presented as a table of potential bacterial markers, a bar chart of the LDA value distribution, and an evolutionary branch map. The Mann Whitney U test was used to analyze significant differences between groups (13). Microbiota function was predicted using the latest version of Phylogenetic Investigation of Communities by Reconstruction of Unobserved States (PICRUSt) 2 (13). The results of gene functional annotation of the microbiota from databases, including COG, EC, KO, PFAM, and TIGRFAM profiling were obtained, and then statistical analysis of metagenomic profiles (STAMP) (14) was used for differential analysis to obtain significantly different gene functions between groups. In all these analyses, Fisher_test was used for between-sample statistics, with a *p* < 0.05 considered statistically significant. Statistics of multiple replicate samples were performed using the rank sum test, with Wilcox_test for two-group comparisons, Kruskal_test for multi-group comparisons, and *p* < 0.05 considered statistically significant.

## 3 Results

### 3.1 Distribution of gut microbes

IHC analysis showed that LPS was mainly distributed in the feces found on the surface of intestinal tissues and epithelial cells and macrophages within the tissues (**Figure 1C**).

### 3.2 Influence of sampling site and cleaning on microbial detection

**Figure 2:**
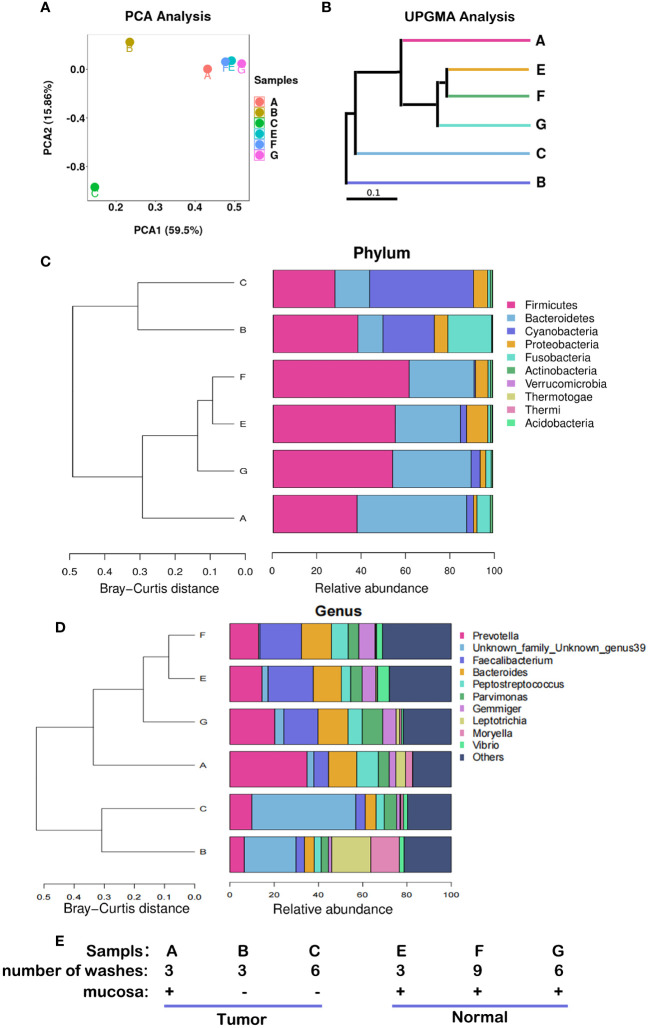
Microbiota abundance with different sampling sites and cleaning conditions. These results were obtained from the analysis of six samples from the 12th patient with different treatment conditions. **(A)** PCA, the more similar the species composition of the samples, the closer they are in the PCA plot. **(B)** UPGMA analysis, the shorter the branch length between samples, the higher the similarity of the two samples. **(C)** Microflora distribution at the phylum level. **(D)** Microflora distribution at the genus level. **(E)** Profiles of the samples. PCA, principal component analysis; UPGMA, Unweighted Pair-Group Method with Arithmetic Mean.

The compositions of microbiota were different among the six samples from the 12^th^ patient (**Figure 2**). Samples E, F, and G were adjacent normal tissue samples (equivalent to part ④ in **Figure 1A**) containing the contaminated intestinal mucosa, which were washed different times as shown in **Figure 2E**. Their respective species compositions are similar. Sample A was the tumor tissue sample (equivalent to part ① in **Figure 1A**) containing the contaminated intestinal mucosa. Sample B (equivalent to part ② in **Figure 1A**) and sample C (equivalent to part ③ in **Figure 1A**) had the mucosa removed and different washing numbers (**Figure 2E**). There was little difference between the bacterial composition of A and that of E, F, and G (**Figures 2A, B**), indicating that their microbiomes were similar. However, there were significant differences between the species compositions of A and B or C (*p* < 0.05). Firmicutes and Bacteroidetes were the dominant species in E, F, and G, whereas the relative abundance of Cyanobacteria was dominant in B and C (**Figure 2C**). At the genus level, the abundance of *Prevotella, Faecalibacterium*, and *Bacteroides* were reduced in B and C compared to that in the other samples, whereas *Unknown_family_Unknown_genus39* and *Vibrio* were present, and the relative abundance of *Leptotrichia* was increased in TT compared to that in NT (**Figure 2D**).

### 3.3 Tumor cell-associated microbiota obtained with cell enzymatic digestion combined with microbial detection

#### 3.3.1 Quality control of sample sequencing data

5R-16s RNA gene analysis was performed on 44 samples from 11 patients with CRC, including CRC tumor tissues (TT), normal tissues adjacent to CRC (NT), tumor cells (TC), normal cells (NC). TC and NC were obtained through separation and extraction of TT and NT tissues using enzymatic digestion. A total of 4,606,353 raw reads (> 100,000 reads per sample) and 4,589,247 clean reads (≥ 99588 reads per sample) were obtained from 44 samples, with an average Q20 of 98.71% and Q30 of 96.19% (**Supplementary Exl S2**).

#### 3.3.2 alpha diversity analysis

**Figure 3:**
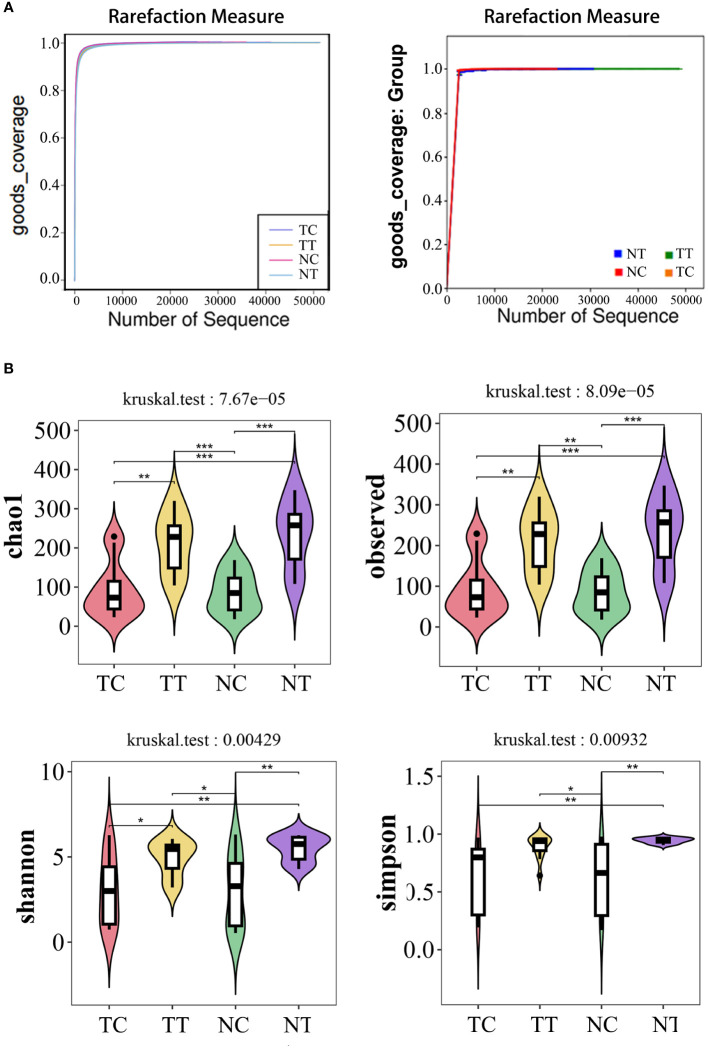
Alpha diversity analysis. Alpha diversity reflects species richness and evenness, as well as sequencing depth. **(A)** Rarefaction curves of good index. When the curve tends to be flat, it indicates that the amount of sequencing data is rich enough, and further increasing the amount of data will contribute little to the discovery of more species. Good coverage reflected the flora coverage of the sample. **(B)** violin plot: the Chao1 and Observed analysis reflected the species richness of the samples, the Simpson and Shannon analyses are mainly reflected the richness and evenness of species. *: *p< 0.05*, **: *p< 0.01*, ***: *p< 0.001*.

In the alpha diversity analysis, the alpha rarefaction curve showed a reasonable sequencing depth and a good value of 1 (**Figure 3A**), indicating complete coverage of microbiota sequencing. Chao1 and observed_species were used to estimate the number of species in a community. The results showed that the number of species in the cell groups after using the enzymatic digestion method was significantly lower than that in the tissue groups (*p* < 0.05). The ratio of the microbe number in cells to that of tissues was higher in tumors than that in normal tissues (**Table 1**; **Figure 3B**) (Additional rarefaction curves are shown in **Supplementary Figure S3**).

**Table 1:** alpha_diversity_statistics.

alpha_diversity	TC	NC	TT	NT
goods_coverage	1.00±0.00	1.00±0.00	1.00±0.00	1.00±0.00
chao1	93.06±70.65	86.09±52.34	210.29±75.97	236.37±85.83
observed_species	92.91±70.41	86.09±52.34	209.82±75.67	235.91±85.68
shannon	3.02±2.03	2.94±2.15	4.95±1.05	5.52±0.75
simpson	0.63±0.32	0.59±0.33	0.89±0.10	0.94±0.02

#### 3.3.3 Beta diversity analysis

**Figure 4:**
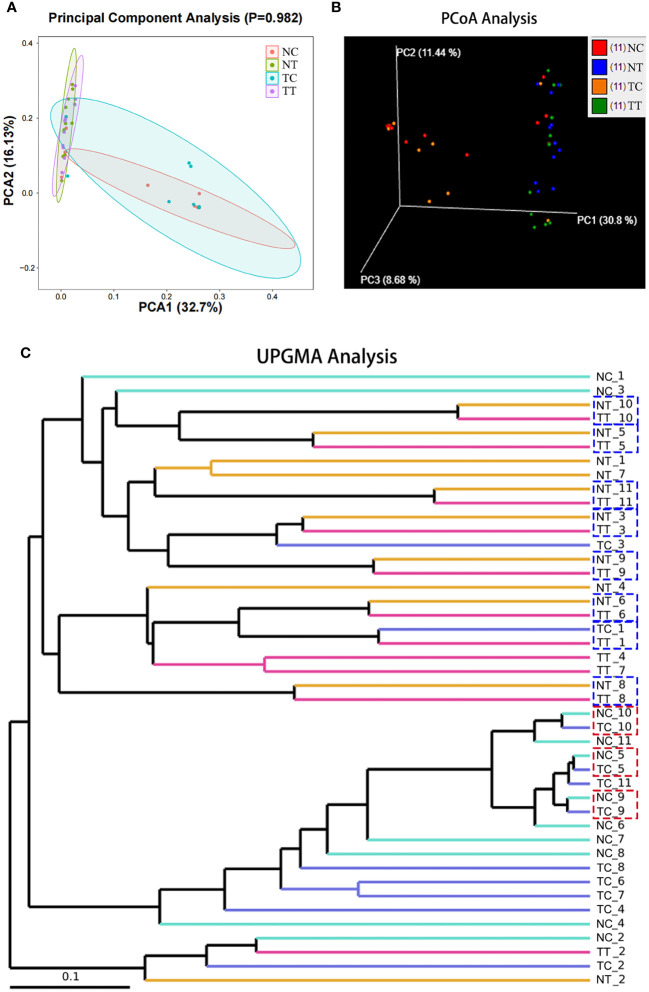
Beta diversity analysis. Beta diversity reflects species differences between groups. **(A)** The more similar the species composition of the samples, the closer they are in the PCA plot. **(B)** A closer distance between two points indicates a smaller difference in community composition in the PCoA plot. **(C)** The shorter the branch length between samples, the more similar two samples are. PCA, principal component analysis; PCoA, principal coordinate analysis.

Beta diversity reflects species differences between environmental communities. Beta and alpha diversities constitute the overall diversity or biological heterogeneity of an environmental community. Beta diversity analysis mainly measures the differences between samples using PCA, PCoA, the Unweighted Pair-Group Method with Arithmetic mean (UPGMA), ANOSIM, and other methods. The results of PCA and PCoA showed no obvious differences between the TT and NT groups or between the TC and NC groups, but there were differences between the cells and tissues (**Figures 4A, B**). The UPGMA analysis results show that the microbiomes of eight out of 11 patients had few differences between tumor and normal tissue samples. However, after enzymatic hydrolysis, only the microbiomes of the tumor and normal cell samples of three patients were similar, whilst the microbiomes of the cell samples from most patients showed obvious differences (**Figure 4C**). This indicates that the data for each group were consistent and that the enzymatic hydrolysis of cells was effective. The ANOSIM results showed a significant difference in microbiota composition between the cell and tissue groups (*R > 0*, *p < 0.05*) (**Table 2**).

**Table 2:** anosim_result.

Method name	*R* statistic	*p*_value	Groups
bray_curtis	0.56	0.00	NC_vs_NT
bray_curtis	-0.03	0.72	TC_vs_NC
bray_curtis	0.47	0.00	TC_vs_TT
bray_curtis	-0.07	0.93	TT_vs_NT

#### 3.3.4 Analysis of microbiota

**Figure 5:**
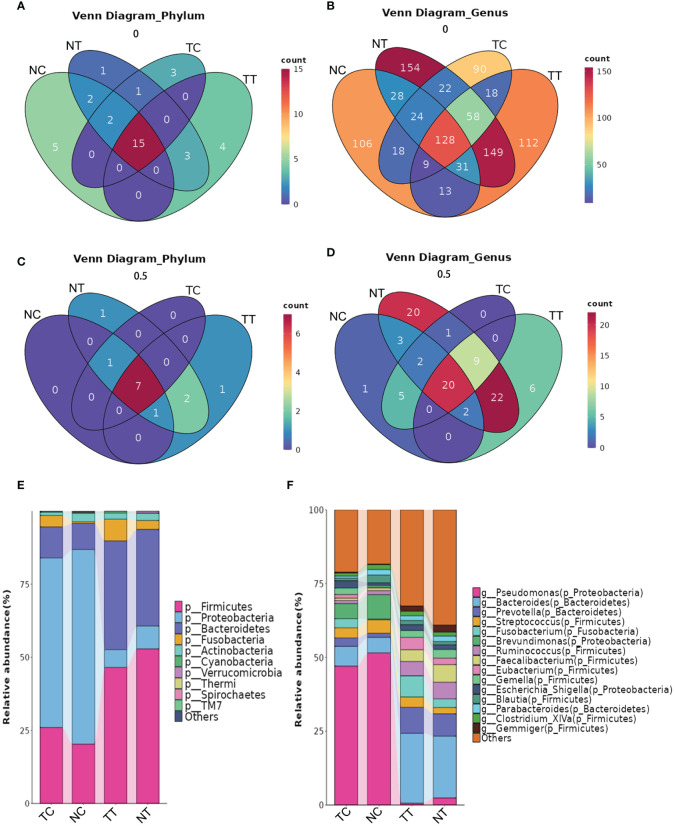
The distribution of microbiota among the groups at the phylum and genus levels. **(A)** Venn analysis of all species at the phylum level. **(B)** Venn analysis of all species at the genus level. **(C, D)** Venn analysis of species present in more than half of the samples at the phylum and genus levels. **(E, F)** Column stacking charts of species abundance at the phylum and genus levels. TC, TT, NC, and NT are the four groups. NC, normal cell; NT, normal tissue; TC, tumor cell; TT, tumor tissue.

The distribution of the microbiota differed among the groups at the phylum and genus levels. A total of 22 phyla and 518 genera were detected in the TT samples, 24 phyla and 594 genera in the NT samples, 21 phyla and 367 genera in the TC samples, and 24 phyla and 357 genera in the NC samples (**Figures 5A, B**). Microbial species that were present in more than half of the 11 patient samples were further analyzed and found that eleven phyla and 59 genera were detected in TT samples, 12 phyla and 79 genera in NT samples, 8 phyla and 37 genera in TC samples, and 9 phyla and 33 genera in NC samples (**Figures 5C, D**). At the phylum level (**Figure 5E**), Firmicutes, Bacteroidetes, and Proteobacteria were the most abundant in the tissue samples, followed by Actinobacteria and Fusobacteria. Notably, Firmicutes and Bacteroidetes were highly abundant in tissues, whereas Proteobacteria were the most abundant in the treated cell samples. The abundance of Firmicutes was lower in the tumor tissues than that in the normal tissue groups but was higher in the tumor cell groups. There was no significant difference in Actinobacteria abundance between the tumor tissue and normal tissue groups (*p > 0.05)*. At the genus level, *Pseudomonas, Bacteroides, Brevundimonas*, and *Lactobacillus* were predominant in the TC group. *Pseudomonas, Brevundimonas, Bacteroides*, and *Streptococcus* were the dominant bacteria in the NC group, whereas *Bacteroides, Prevotella, Fusobacterium*, and others were the dominant bacteria in the TT group. The dominant bacteria in the NT group were *Bacteroides, Prevotella*, and *Faecalibacterium* (**Figure 5F**). The percentages of the top 10 species are shown in the pie chart in **Supplementary Figure S4**.

#### 3.3.5 Analysis of biomarkers

**Figure 6:**
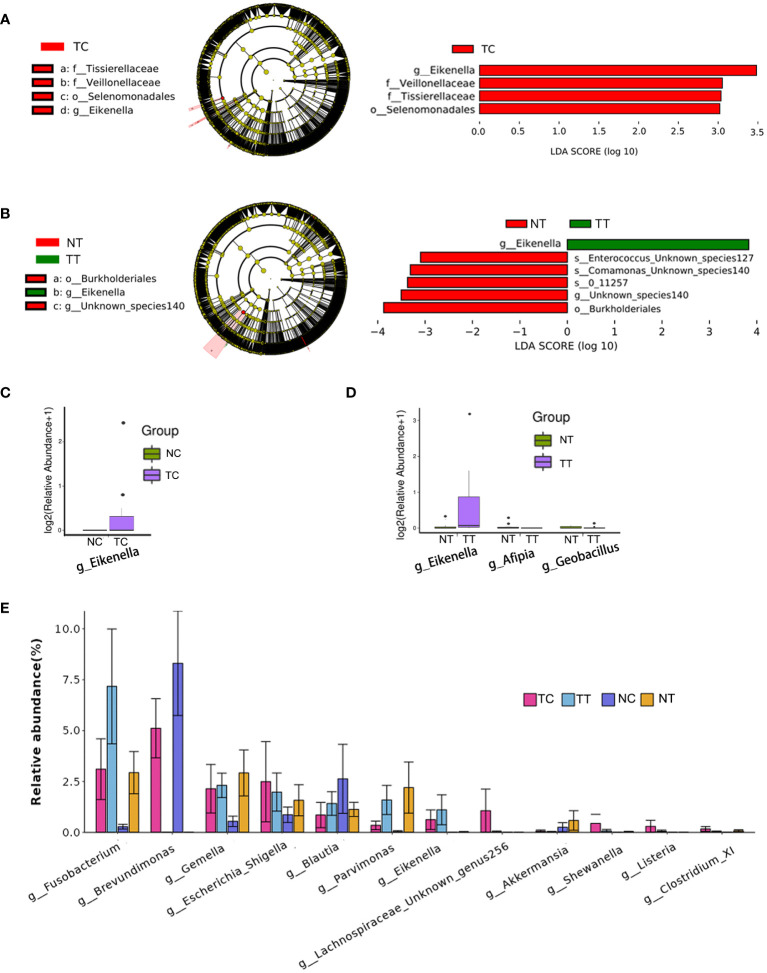
Analysis of biomarkers. LEfSe analysis is the most common method for differential analysis of microbiota. LDA*>3.0, p< 0.05* were used as a threshold for discriminative features. **(A, B)** Cladogram and LDA score bar graph. **(C, D)** Mann–Whitney U test analysis. **(E)** Abundance maps of some of the different species. TC, TT, NC, and NT are the four groups. LEfSe, linear discriminant analysis effect size; NC, normal cell; NT, normal tissue; TC, tumor cell; TT, tumor tissue.

To identify differences in the compositions of the microbiota among groups, the LEfSe algorithm was used. Using LDA>3.0 as a threshold for discriminative features, as shown in the cladogram and LDA score bar graph (**Figures 6A, B**), we identified significant differences in the abundances of Veillonellaceae and Tissierellaceae at the family level and *Eikenella* at the genus level (*p < 0.05*), while these were more abundant in the TC group than in the NC group. Additionally, *Eikenella* was more abundant in TT than in NT. Identical results were obtained using the Mann–Whitney U test (**Figures 6C, D**), and *Eikenella* remained significantly more abundant in the TT and TC groups than in the normal groups (*p < 0.05*). The results of TC versus TT and NC versus NT are shown in **Supplementary Figures S5~10**. Comparison among the four groups revealed that the abundance of some bacteria species was higher in the tumor groups than that in the normal groups, such as *Fusobacterium, Eikenella, Shewanella, and Listeria*, whereas the abundance of *Akkermansia* was lower in the tumor group than that in the normal group. The ratios of some bacteria species in cells to that in tissues, such as *Gemella, Escherichia, Shigella*, and *Blautia*, were remarkable changed (**Figure 6E**).

#### 3.3.6 Predictive functional profiling of microbiota

**Figure 7:**
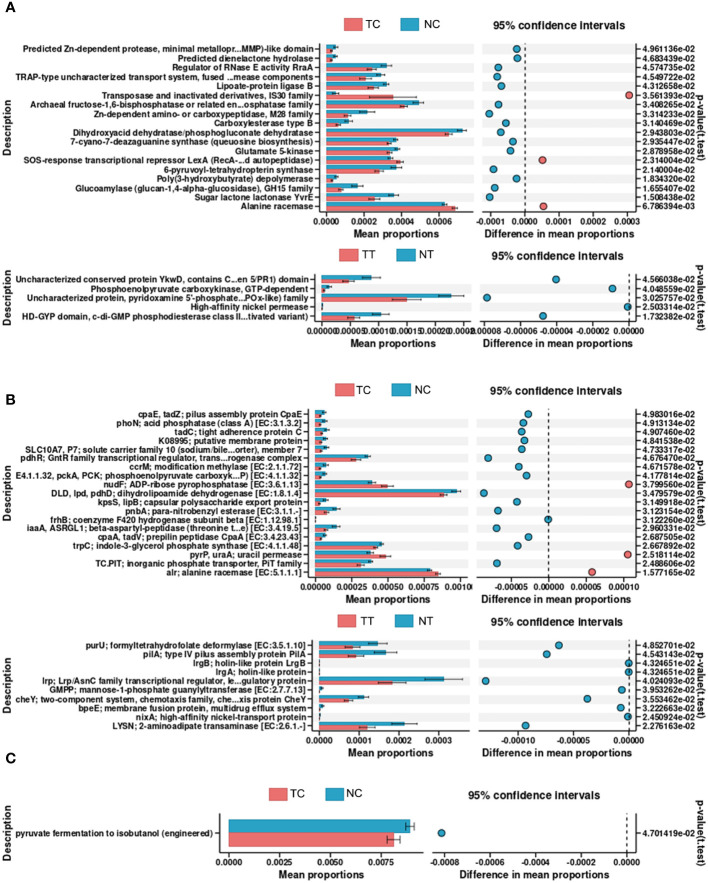
Predictive functional profiling of microbiota. PICRUSt2 was performed to explore the potential functional profiling of CRC microbiota from the four groups. **(A)** COG database results. **(B)** KO database results. **(C)** Pathway database results. TC, TT, NC, and NT are the four groups. CRC, colorectal cancer; NC, normal cell; NT, normal tissue; TC, tumor cell; TT, tumor tissue.

PICRUSt2 was used to explore the potential functional profiles of the CRC microbiota from the four groups. The COG results showed significant differences in the levels of predicted Zn-dependent protease, minimal metalloprotease (MMP)-like domain, dienelactone hydrolase, regulator of RNase E activity, and RraA between the TC and NC groups (*p < 0.05*); Uncharacterized conserved protein YkwD, contains CAP (CSP/antigen 5/PR1) domain, phosphoenolpyruvate carboxykinase, GTP-dependent uncharacterized protein, pyridoxamine 5’-phosphate oxidase (PNPOx-like) family, high-affinity nickel permease, HD-GYP domain, and c-di-GMP phosphodiesterase class II (or its inactivated variant) levels were significantly different between the TT and NT groups (*p < 0.05*) (**Figure 7A**). KO results showed that significant differences in the levels of pilus assembly proteins, CpaE, phoN, acid phosphatase (class A), tight adherence protein C, and putative membrane proteins between the TC and NC groups (*p < 0.05*); formyltetrahydrofolate deformylase, type IV pilus assembly protein PilA, and holin-like protein LrgB levels were significantly different between the TT and NT groups (*p < 0.05*) (**Figure 7B**). The pathway analysis results showed that only the function of pyruvate fermentation to isobutanol (engineered) was significantly different between the TC and NC groups (*p < 0.05*) (**Figure 7C**); there was no difference in the functions between the TT and NT groups. The results for other groups are provided in **Supplementary Figures S11, S12**.

## 4 Discussion

The intestinal microecology plays an important role in the occurrence and development of CRC. In recent years, the development of sequencing technology and bioinformatics has enabled the study of the composition, metabolism, and metagenome of intestinal microecology (14). The gut microbiota has various effects on the transformation process, progression, and response of tumors to immunotherapy. In addition to multiple functions, such as maintaining the epithelial barrier, immune response, digestive function, and regulating neurotransmitters, the gut microbiota are essential for maintaining homeostasis. Changes in the gut microbiota may interfere with homeostasis, leading to dysregulation of the flora (15). Microbiota dysregulation manifests as changes in composition, decreases in diversity, and increases in potential pathogenic bacteria (16, 17). Dysregulated intestinal microecology has “pre-carcinogenic properties” and contributes to the formation of a tumor-like microenvironment. Intestinal epithelial function impairment, pathogen recognition dysfunction, and abnormal immune response accompanied by the dysregulation of the microbiome lead to a severe reduction in intestinal resistance to colonization by pathogenic microorganisms, thus making it easier for pathogenic bacteria to invade intestinal epithelial tissues and cells (18). Additionally, the dysregulation of microbiota can promote the proliferation, invasion and metastasis of tumor cells by increasing the release of endotoxin and mediating the M2 polarization of TLR4-dependent macrophages (19, 20). Furthermore, dysregulated microbiota can have a significant impact on chemotherapy efficacy by affecting drug absorption, decomposition, and toxicity, thus affecting the development and prognosis of CRC (15).

While many studies have revealed a strong relationship between gut microbiota and CRC, most have focused on fecal microbiota and their metabolites. In 2020, Nejman et al. (5) studied 1,526 tumors and adjacent normal tissues from seven cancer types: breast, lung, ovarian, pancreatic, melanoma, bone, and brain tissue tumors and found that tumor microbiota are composed of tumor-specific intracellular bacteria. In addition, intracellular bacteria can cause cells to become cancerous by hybridizing their own DNA with that of the host cell (21). Host cells infected with bacteria undergo changes in metabolism, exhibiting a “Warburg-like metabolism” similar to that of cancer cells (22). After bacteria enter cancer tissues or cells, they not only affect chemotherapy efficacy through drug transformation, but also antibiotic resistance through immune evasion (23). Bacteria colonize tumors in humans, proliferate within them, and modulate immune function, ultimately affecting the survival of patients with cancer and their response to treatment (24). In 2022, Fu et al. found that intracellular bacteria enhanced the resistance of circulating tumor cells to fluid shear stress by reorganizing the cytoskeleton, improving the survival ability of host cells, and thus promoting tumor metastasis (25). In summary, intratumoral microbiota play an important and direct role in inducing tumor occurrence, altering cell metabolism, promoting tumor metastasis, and enabling the tumor to evade immune surveillance. The in-depth study and subsequent regulation of intratumoral microbiota may aid the discovery of novel treatment options for patients with cancer (5).

However, given the low microbial biomass of tumors, characterization of tumor microbiome remains a challenge. Tumors have a high host-to-bacterial DNA ratio, which makes the identification of tumor-associated bacteria extremely complex (26). Nevertheless, with the improvements in new-generation analytic tools, there has been some progress in the study of intratumoral microbiomes in recent years. Moreover, it has now been proven that each cancer subtype has a unique microbiome, characterized by bacterial communities with specific metabolic functions (5, 26).

However, in contrast to most tumors, such as breast cancer, which have no direct connection with the external environment, CRC tumor microbes are contaminated by fecal microbes owing to the structure of the gut folds and villi. The low microbial biomass within the tumor, the large and complex intestinal microbiome, and dealing with contamination, are all challenges in detecting tumor-associated microbiota in CRC. These difficulties have limited research on intratumoral microbes in the tumors that are directly related to the external environment. In a study by Nejman et al., they included 22 colorectal tumors to enrich their analysis. Unlike that of other cancers, such as lung and ovarian cancer, the Jaccard index of bacterial species profiles was similar between CRC and normal tissues, and bacteria belonging to the Firmicutes and Bacteroidetes phyla were the most abundant species in colorectal tumors (5). These results were consistent with those obtained in our tissue-level study and a previous study (27). In another study, Dohlman et al. distinguished tissue-resident microbiota from contaminants. It was found that the Firmicutes and Bacteroidetes were the most dominant phyla in CRC tissues, and bacteria in CRC tissues were enriched for mucosa-related species (28). In the four major gastrointestinal cancer microbiome studies, no differences in microbial community profiles were observed between tumor and normal samples (29).

LPS is a component of the cell wall in Gram-negative bacteria. Most intestinal bacteria are Gram-negative, such as *Escherichia coli*. Bacteria were mainly distributed in the feces found on the surface of intestinal tissues and in epithelial cells and macrophages within the tissues. This is consistent with the findings of a previous report (4). We attempted to avoid contamination by intestinal fecal bacteria through cleaning the sampling sites and controlling their location. The effects of different sampling sites and number of washes on the detection of CRC intratumoral microbiota were compared. The results showed that different sampling sites and number of washes affected microbiota detection. Although cleaning methods can be controlled, sampling sites varied and led to different microbial distributions. Carcinogenesis is related to the bacterial composition inside the intestinal epithelial cells; however, in the normal intestinal tissues, the contamination of fecal bacteria cannot be avoided due to the tissue structure. Therefore, fecal bacterial contamination cannot be avoided by controlling or cleaning the sampling site in sampling normal tissues.

We established a new method for isolating tumor cell-associated microbiota using cell enzymatic digestion. First, we destroyed the gut folds and villus structures by rough shearing and cell enzymatic hydrolysis to exclude fecal bacterial contamination. In this process, we chose dispase and collagenase, instead of trypsin, in order to destroy the intestinal fold and villus structure, whilst preserving the integrity of the cells. Then, we used a 40-μm cell sieve to filter out the undigested tissue blocks to further avoid contamination of fecal bacteria in the tissue sample. After that, the collected single cell suspension was separated repeatedly with a centrifugal force of 300 x g, to remove the “passing bacteria” where the binding between the cells are bacteria is weak. This process helps to reduce fecal bacterial contamination. The final microbiome consisted mainly of bacteria from the cells or those that colonized the cells.

Finally, 5R-16s rRNA gene sequencing was used to analyze and compare the differences in CRC-associated microbiota of a total of 44 conventional and enzymatically treated samples from 11 patients. The sequencing results of the tissue samples were consistent with those of previous studies (5, 27). However, different results were obtained for the cell samples treated with enzymatic hydrolysis. In contrast to the that at the tissue level, Proteobacteria were the most abundant species in the cell samples. The abundance of the Firmicutes phyla was lower in the tumor tissues than that in the normal tissues but was higher in the cell groups. The proportion of Actinobacteria also differed between the two groups. The cell enzymatic digestion method may reduce the contamination of fecal bacteria, enabling low biomass intratumoral microbiota to be obtained. This was also confirmed at the genus level, where more differential bacteria were found by comparing the abundance and tumor/normal ratios of species.

Combining the detection results from tissues can also predict bacterial distributions. When the biomass of a species is high in both tumor tissues and cells, the bacteria are enriched and have invaded the tumor cells. If there is no difference in the species abundance between the tumor and normal tissues, the intracellular biomass increases, indicating that the bacteria have invaded the tumor. Conventional detection methods can only detect the enrichment of bacteria in tissues, but not the movement of bacteria.

Cell enzymatic digestion can also be combined with microbial culture to detect tumor cell-associated microorganisms, which may be more accurate than tissue culture and narrows the scope of future research. However, owing to the limitations of laboratory conditions, this approach has not been investigated and requires further research. There is a scarcity of research and skillset for tumor research in the microbial field. Therefore, it is difficult to conduct further timely research and comparisons between studies. Due to the difficulty of acquiring clinical biopsy tissues, the sample size is relatively small. Further studies will increase the sample size and expand this study, including using animal models to explore further research, which may identify microbial targets and provide help for future CRC diagnosis and intervention.

This study provides a new method to detect CRC cell-associated microbiota. However, our study has some limitations: first, the sample size is small. A total of 12 patient samples were used and only 11 patient samples were used for full analyses. Second, the method of isolating tumor-associated microbiome was the first of its kind that has not been used by other investigators, the reproducibility of which needs to be validated in another cohort of samples. Third, fecal microbiome were not analyzed using the same 5R-16s rRNA-seq because the abundance of fecal microbiome was way more than the tumor-associated microbiome. Future studies shall use more samples and multiple cohorts of patients including at least a testing cohort and a validation cohort to verify the reproducibility of the study. It is desirable to analyze both fecal and tumor-associated microbiomes and compare the microbial populations between them.

## 5 Conclusion

Different sampling sites and number of washes led to different microbiota detection results. The cell enzymatic digestion method can reduce the coverage of fecal bacteria in intestinal tissues to allow low biomass intratumoral microbiota to be obtained. This is a new method for the isolation of tumor cell-associated microbiota, and in combination with microbial composition analysis of tissues can predict the movement of bacteria. It can also provide additional assistance for generating microbial cultures. Thus, this study provides a new method for studying tumor-associated microbiota.

## Data availability statement

The original contributions presented in the study are included in the article/**Supplementary Material**. Further inquiries can be directed to the corresponding authors.

## Ethics statement

All participants provided informed consent, and the study was approved by the Medical Ethics Committee of Chengde Medical University (No. 202215). The studies were conducted in accordance with the local legislation and institutional requirements. Written informed consent for participation in this study was provided by the participants’ legal guardians/next of kin.

## Author contributions

YZZ: Formal analysis, Writing – original draft. YJL: Funding acquisition, Writing – original draft. JYP: Methodology, Writing – original draft. SKJ: Methodology, Writing – review & editing. XYZ: Data curation, Writing – original draft. EHZ: Resources, Writing – review & editing. YHL: Funding acquisition, Writing – review & editing.
