# Supplementary material for: Detection and comparison of tumor cell-associated microbiota from different compartments of colorectal cancer
Source: Front Oncol. 2024 May 21;14:1374769. doi: 10.3389/fonc.2024.1374769 (PMC11148212; doi:10.3389/fonc.2024.1374769)
Supplement: Supplementary file 1 [file Presentation_1.zip › Supplementary Material/Supplementary Material S1.docx]

1. **Primer sequences of five (V2, V3, V5, V6, and V8) regions on the 5R-16s rRNA gene sequencing：**

| V2 | F1: 5'-TGGCGAACGGGTGAGTAA-3'  R1: 5'-AGACGTGTGCTCTTCCGATCTCCGTGTCTCAGTCCCARTG-3'  FF1:5'AATGATACGGCGACCACCGAGATCTACACTCTTTCCCTACACGACGCTCTTCCGATCTTGGCGAACGGGTGAGTAA-3'; |
| --- | --- |
| V3 | F2: 5'-ACTCCTACGGGAGGCAGC-3'  R2: 5'-AGACGTGTGCTCTTCCGATCTGTATTACCGCGGCTGCTG-3'  FF2:5'AATGATACGGCGACCACCGAGATCTACACTCTTTCCCTACACGACGCTCTTCCGATCTACTCCTACGGGAGGCAGC-3'; |
| V5 | F3: 5'-GTGTAGCGGTGRAATGCG-3'  R3: 5'-AGACGTGTGCTCTTCCGATCTCCCGTCAATTCMTTTGAGTT-3'  FF3:5'AATGATACGGCGACCACCGAGATCTACACTCTTTCCCTACACGACGCTCTTCCGATCTGTGTAGCGGTGRAATGCG-3'; |
| V6 | F4: 5'-GGAGCATGTGGWTTAATTCGA-3'  R4: 5'-AGACGTGTGCTCTTCCGATCTCGTTGCGGGACTTAACCC-3'  FF4:5'AATGATACGGCGACCACCGAGATCTACACTCTTTCCCTACACGACGCTCTTCCGATCTGGAGCATGTGGWTTAATTCGA-3'; |
| V8 | F5: 5'-GGAGGAAGGTGGGGATGAC-3'  R5: 5'-AGACGTGTGCTCTTCCGATCTAAGGCCCGGGAACGTATT-3'  FF5:5'AATGATACGGCGACCACCGAGATCTACACTCTTTCCCTACACGACGCTCTTCCGATCTGGAGGAAGGTGGGGATGAC-3'; |

The post-primer of the five variable regions of the 5R 16SrRNA above was RR5, and its sequence was as follows:

RR5:5'CAAGCAGAAGACGGCATACGAGATNNNNNNNNGTGACTGGAGTTCAGACGTGTGCTCTTCCGATCT-3'

1. **the software package used for determining alpha and beta diversity measurements**

| PCA | R-3.4.4 | vegan(2.5.4), ggplot2(3.2.0) |
| --- | --- | --- |
| PCoA | R-3.4.4 | ade4(1.7.13), vegan(2.5.4) |
| UPGMA | upgma | 1.1 |
| anosim | qiime2 | 2019.7 |
