# Supplementary figures and images for: Detection and comparison of tumor cell-associated microbiota from different compartments of colorectal cancer
Source: Front Oncol. 2024 May 21;14:1374769. doi: 10.3389/fonc.2024.1374769 (PMC11148212; doi:10.3389/fonc.2024.1374769)

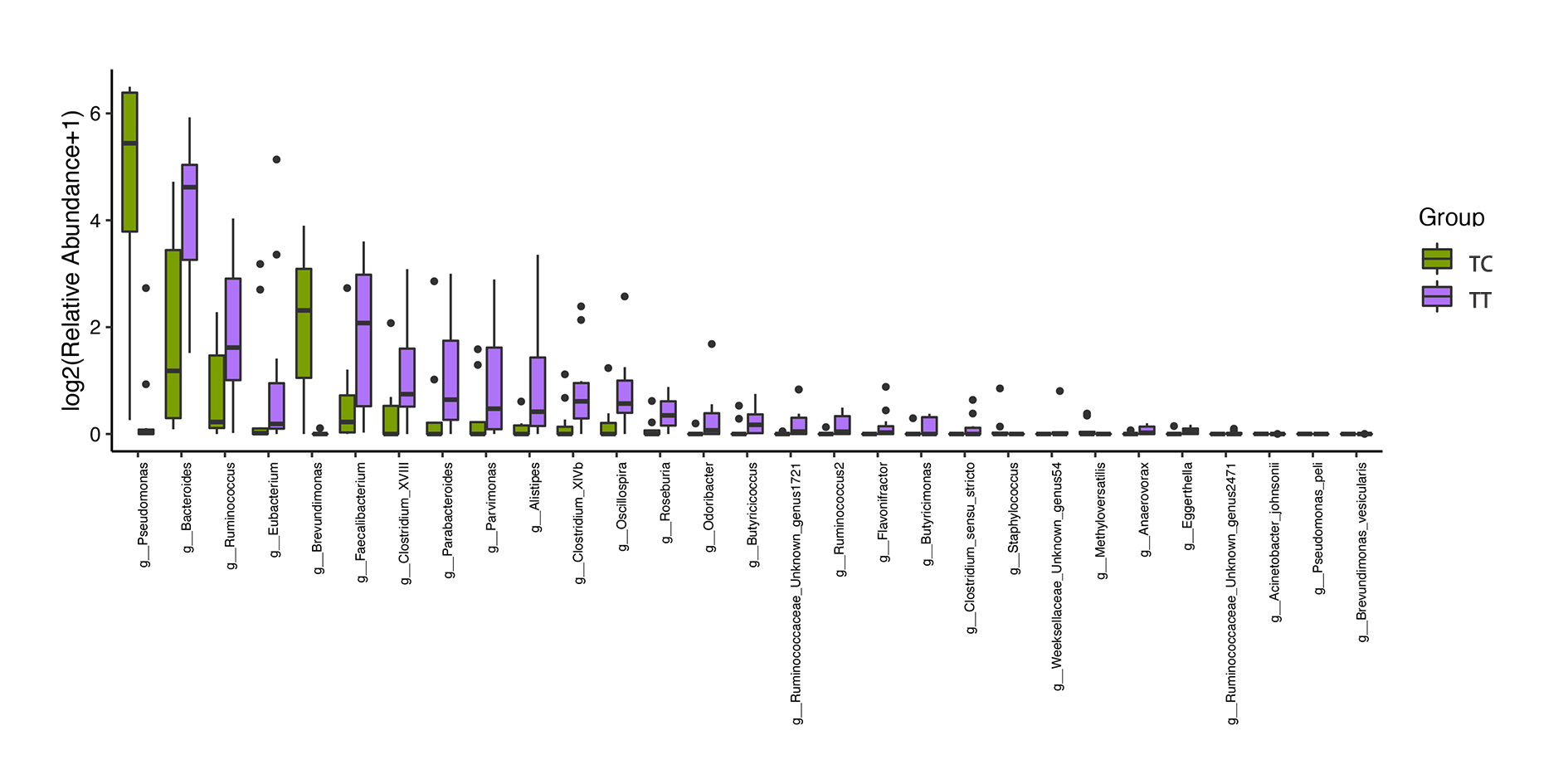

Supplement: Supplementary file 1 [file Presentation_1.zip › Supplementary Material/Supplementary Figure S10-Group.TC_vs_TT_diff_boxplot ┐╜▒┤.tif]

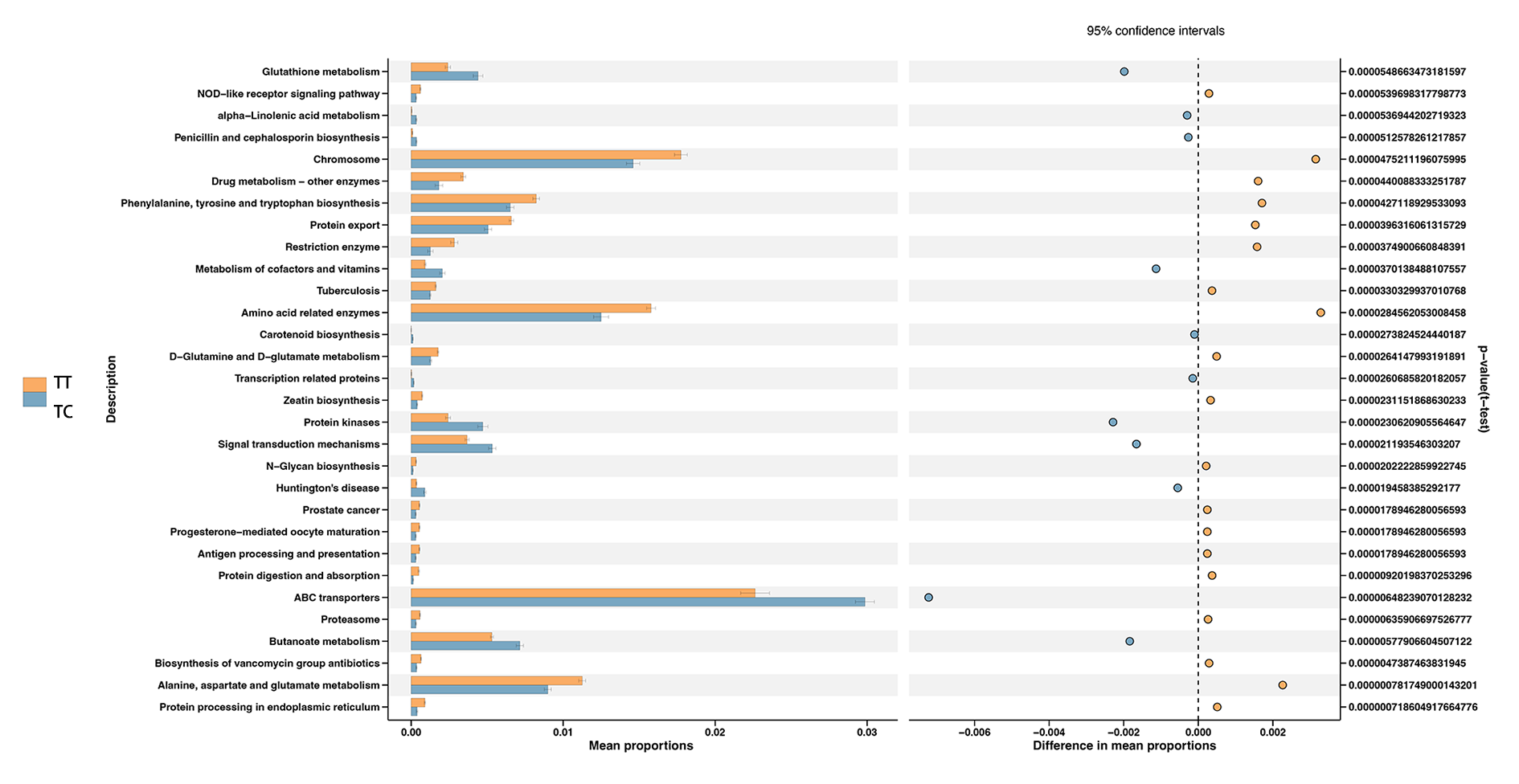

Supplement: Supplementary file 1 [file Presentation_1.zip › Supplementary Material/Supplementary Figure S11-PICRUSt2_KEGG_level3_Group.TC_vs_TT_diff ┐╜▒┤.tif]

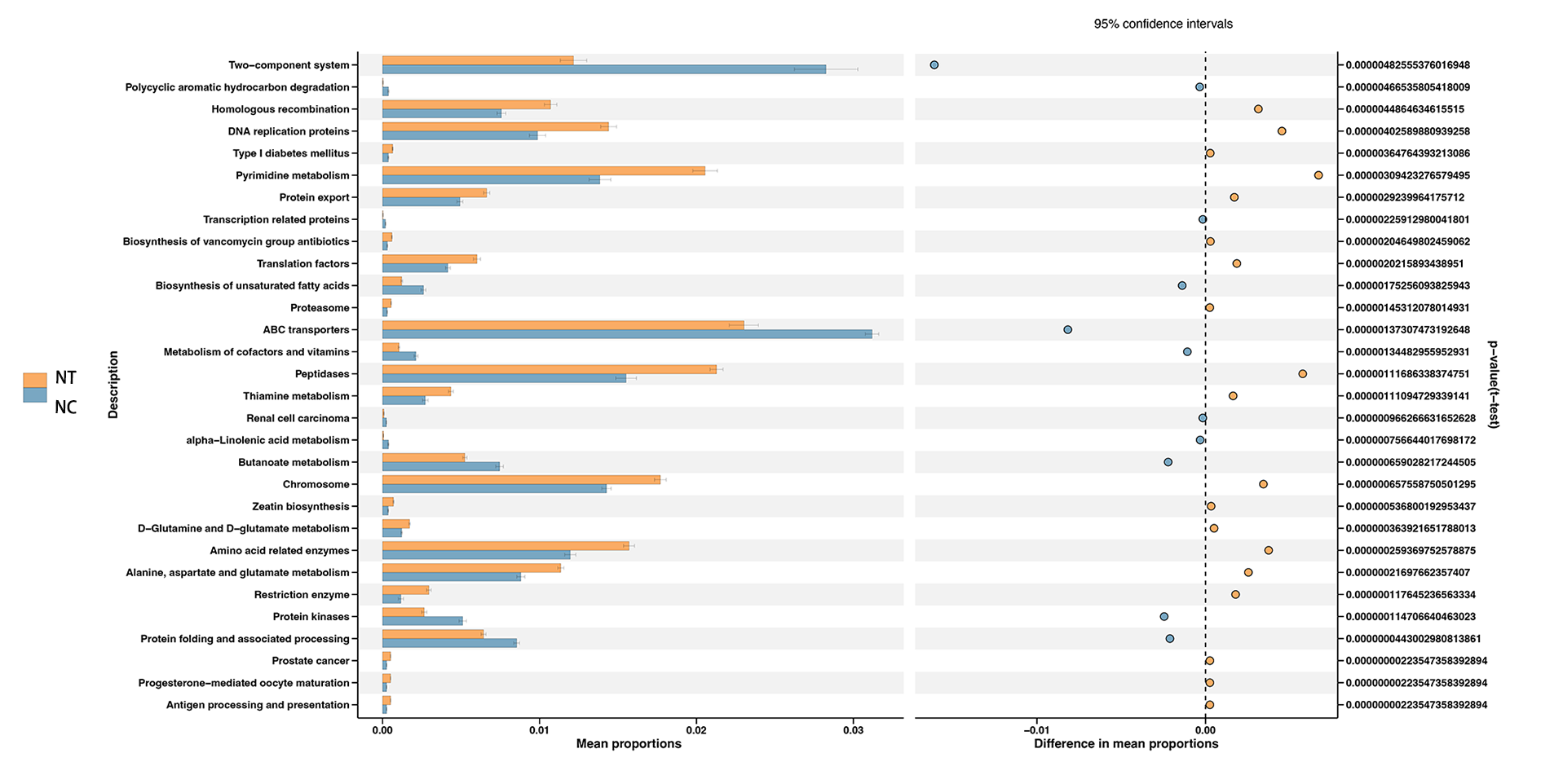

Supplement: Supplementary file 1 [file Presentation_1.zip › Supplementary Material/Supplementary Figure S12-PICRUSt2_KEGG_level3_Group.NC_vs_NT_diff ┐╜▒┤.tif]

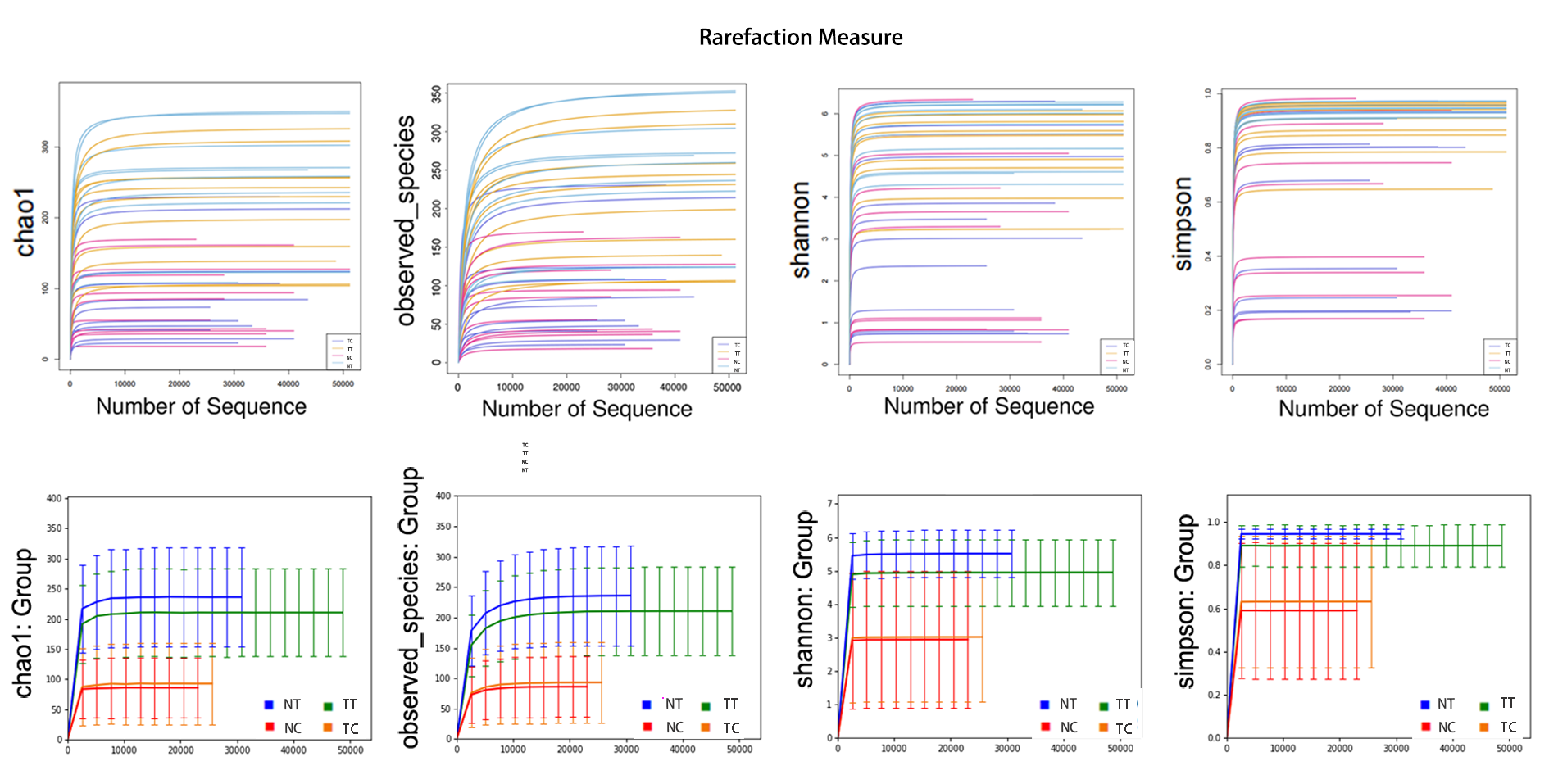

Supplement: Supplementary file 1 [file Presentation_1.zip › Supplementary Material/Supplementary Figure S3-Rarefaction Measure ┐╜▒┤.tif]

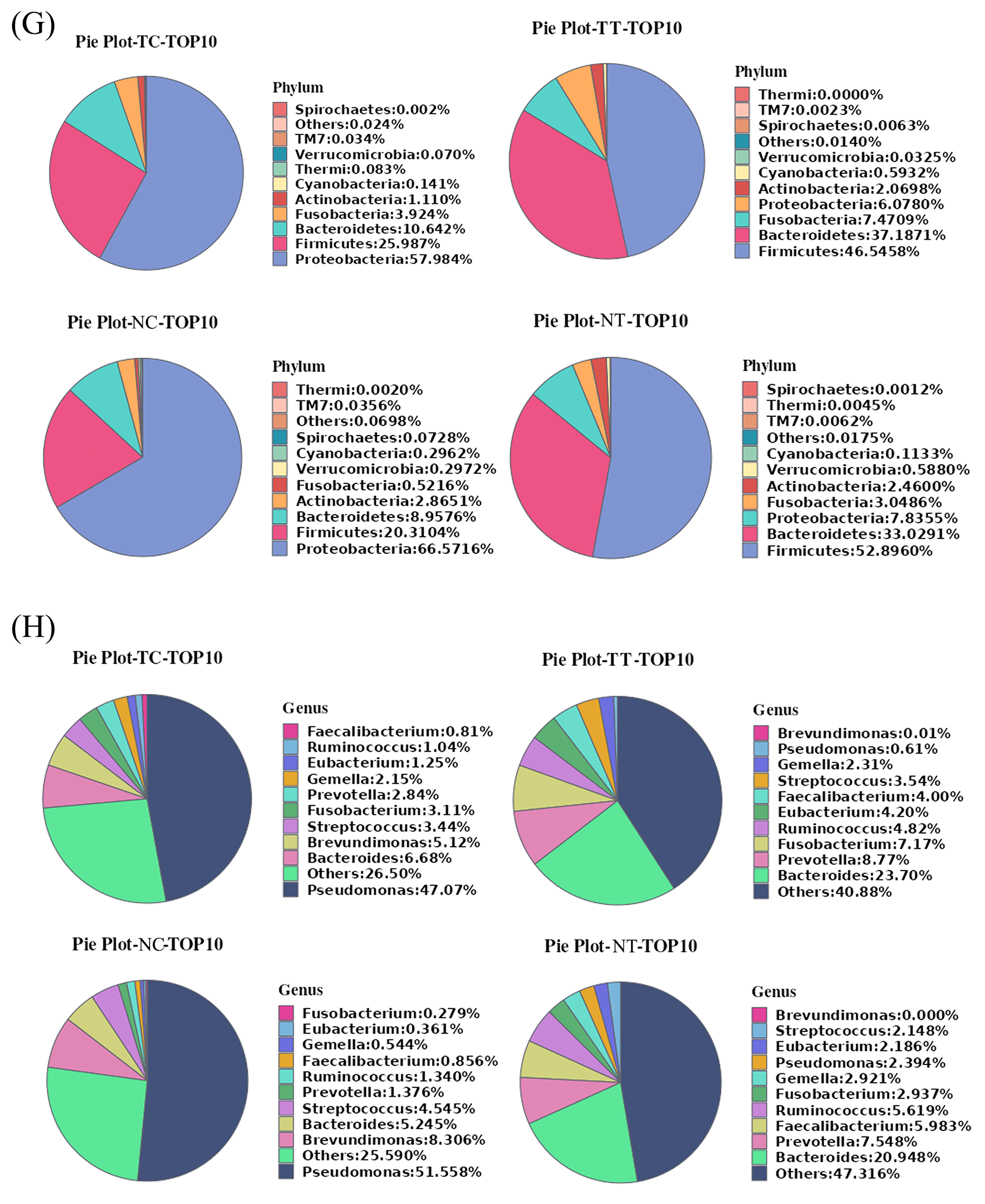

Supplement: Supplementary file 1 [file Presentation_1.zip › Supplementary Material/Supplementary Figure S4-pie chart analysis.tif]

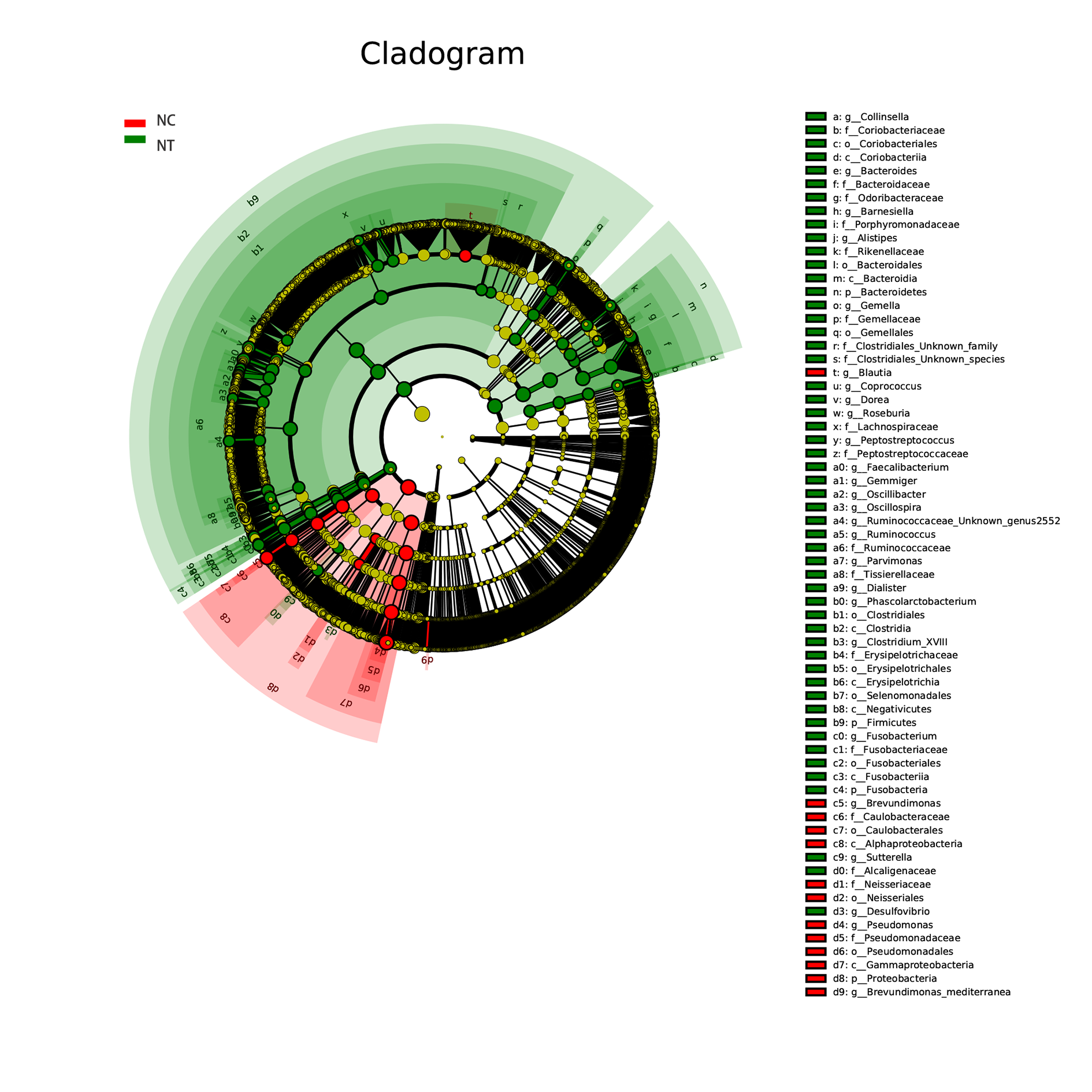

Supplement: Supplementary file 1 [file Presentation_1.zip › Supplementary Material/Supplementary Figure S5-LEfSe_Group_NC_vs_NT.cladogram ┐╜▒┤.tif]

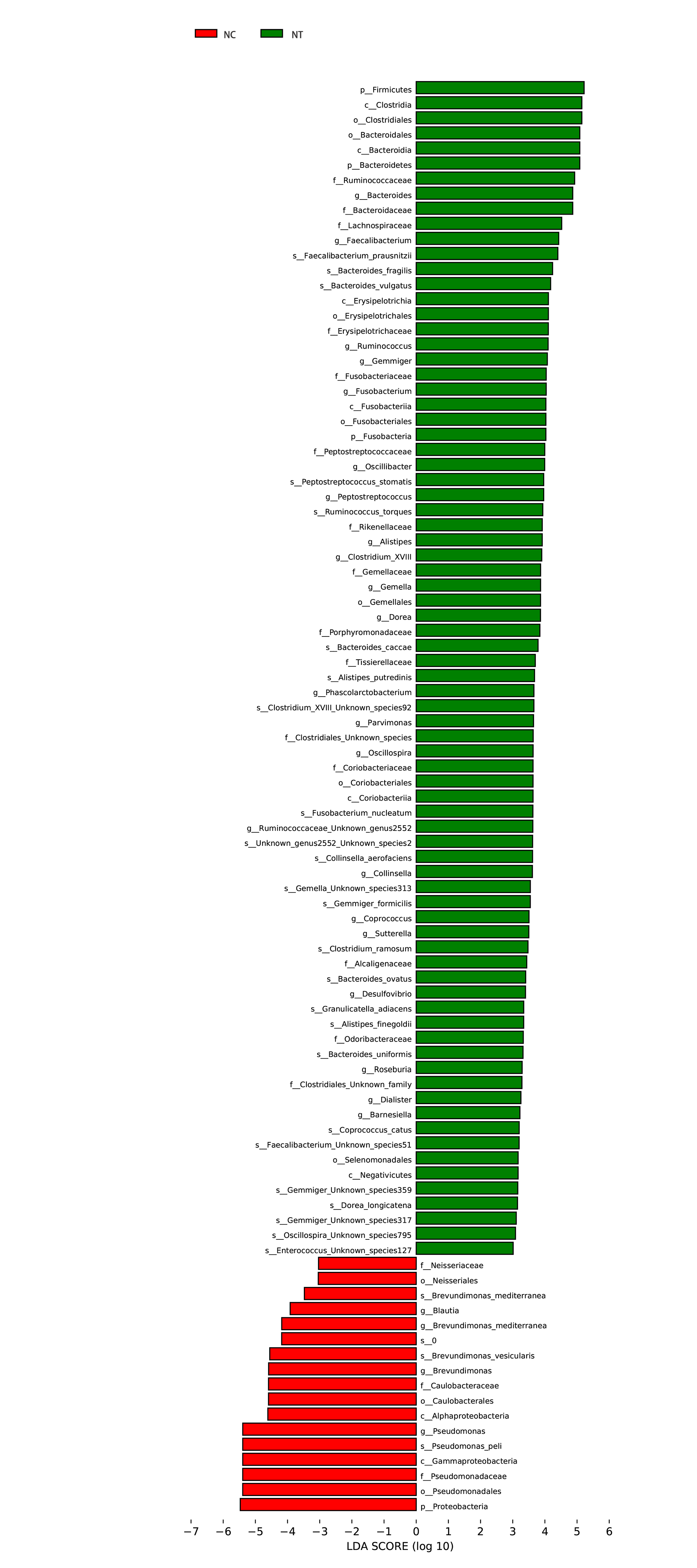

Supplement: Supplementary file 1 [file Presentation_1.zip › Supplementary Material/Supplementary Figure S6-LEfSe_Group_NC_vs_NT ┐╜▒┤.tif]

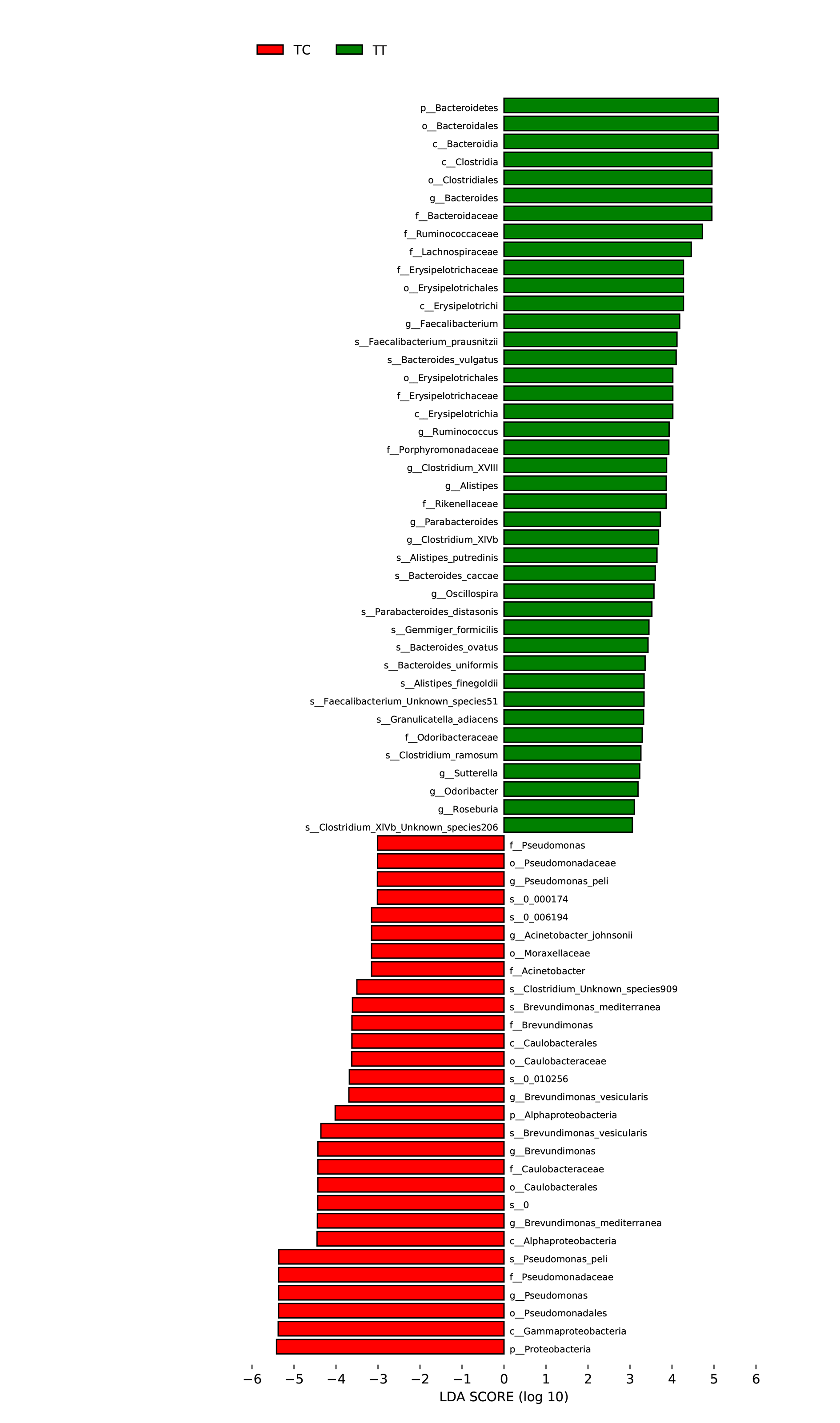

Supplement: Supplementary file 1 [file Presentation_1.zip › Supplementary Material/Supplementary Figure S7-LEfSe_Group_TC_vs_TT ┐╜▒┤.tif]

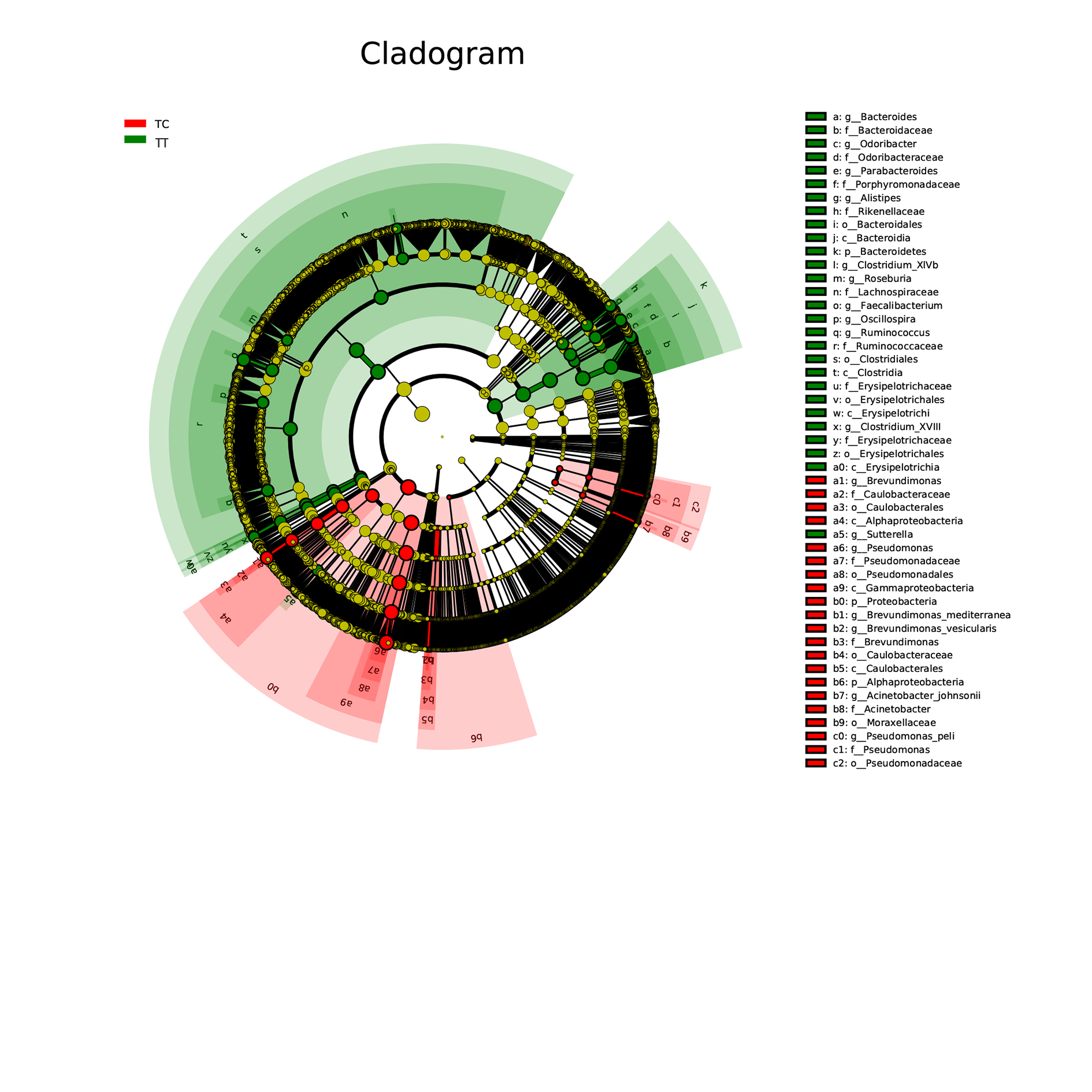

Supplement: Supplementary file 1 [file Presentation_1.zip › Supplementary Material/Supplementary Figure S8-LEfSe_Group_TC_vs_TT.cladogram ┐╜▒┤.tif]

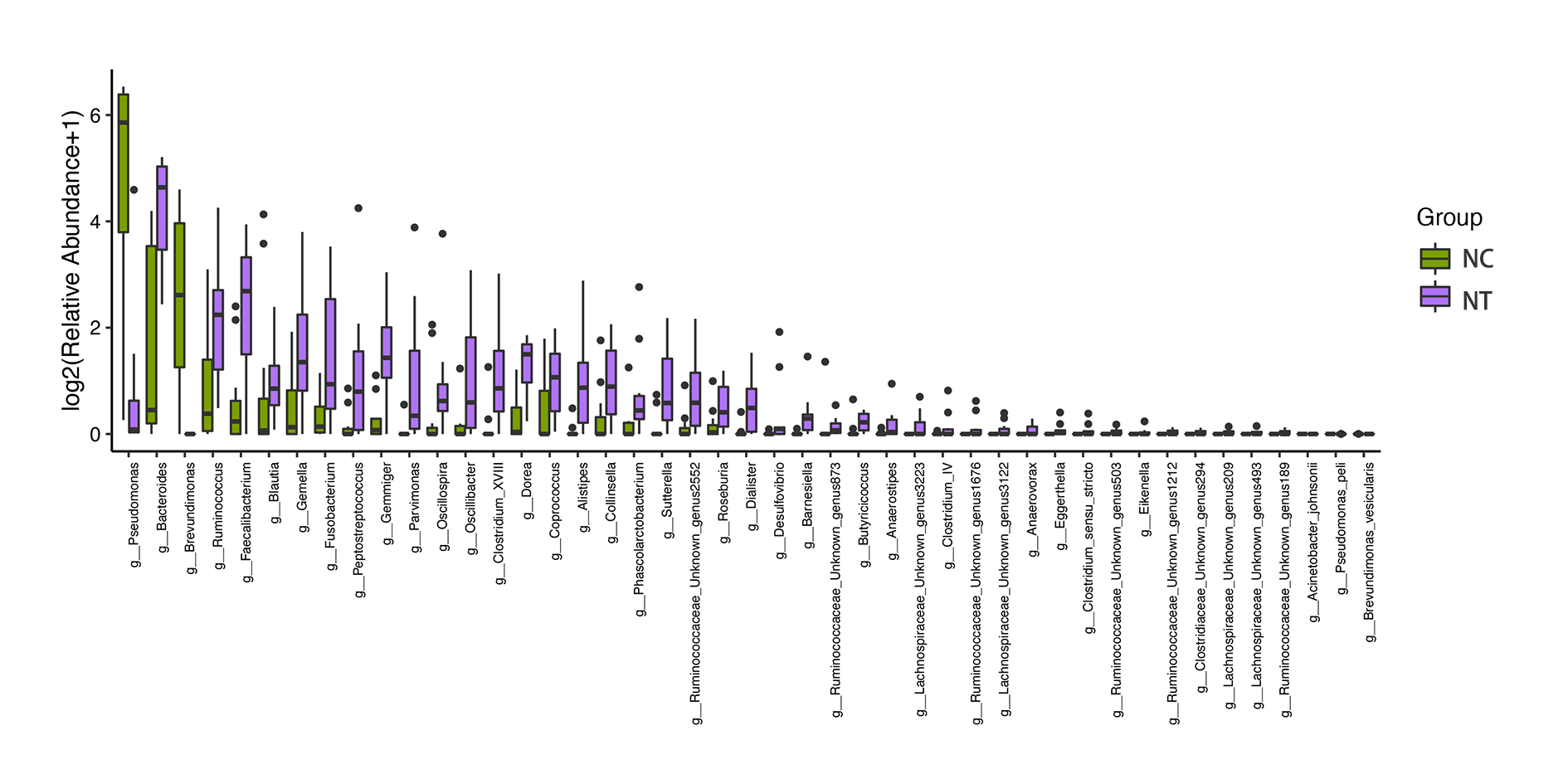

Supplement: Supplementary file 1 [file Presentation_1.zip › Supplementary Material/Supplementary Figure S9-Group.NC_vs_NT_diff_boxplot ┐╜▒┤.tif]
